# Fatigue in amyotrophic lateral sclerosis and correlated factors

**DOI:** 10.1055/s-0042-1758563

**Published:** 2022-12-19

**Authors:** Mariana Asmar Alencar, Bruna Laura Soares, Marcela Ferreira de Andrade Rangel, Juliana Silva Abdo, Rayane Alves Pereira de Almeida, Caroline Martins de Araújo, Leonardo Cruz de Souza, Gisele de Cássia Gomes

**Affiliations:** 1 Universidade Federal de Minas Gerais, Departamento de Fisioterapia, Belo Horizonte MG, Brazil.; 2 Universidade Federal de Minas Gerais, Programa de Pós-Graduação em Neurociência, Belo Horizonte MG, Brazil.; 3 Universidade Federal de Minas Gerais, Departamento de Medicina Interna, Belo Horizonte MG, Brazil.

**Keywords:** Amyotrophic Lateral Sclerosis, Fatigue, Physical Functional Performance, Esclerose Amiotrófica Lateral, Fadiga, Desempenho Físico Funcional

## Abstract

**Background** Amyotrophic lateral sclerosis (ALS) is a fatal neurodegenerative disease that leads to muscle weakness and paralysis. Fatigue is a disabling symptom, frequently reported in ALS, but remains under-investigated in this population. Thus, an accurate investigation of this symptom and possible associated factors in this clinical condition is needed to assist in the establishment of an adequate treatment approach.

**Objective** To investigate the presence of fatigue in individuals with ALS and possible factors correlated with this symptom.

**Methods** Sixty-five individuals with sporadic ALS participated in the present study. Demographic, clinical, and functional aspects were investigated. Evaluations involved the Fatigue Severity Scale (FSS), ALS Functional Scale (ALSRFS-R), and Quality of Life (QoL) questionnaire (ALSAQ-40). Descriptive and correlation analyses were performed with SPSS statistical program for Windows version 19.0 (IBM Corp., Armonk, NY, USA).

**Results** Among the 65 individuals evaluated, 44.6% ( *n* = 29) presented fatigue based on the FSS. The mean fatigue intensity was 5.4 ± 1.2 and only 10.4% used a specific medication for fatigue. Differences between the groups with and without fatigue were found regarding sex ( *p* = 0.049), pain intensity ( *p* = 0.026), functioning ( *p* = 0.004), disease severity ( *p* = 0.029), and QoL ( *p* = 0.000). Fatigue was correlated with pain intensity (r = 0.425; *p* = 0.001), muscle strength (r = - 0.356; *p* = 0.004), functioning (r = - 0.363; *p* = 0.003), and QoL (r = 0.481; *p* = 0.000). No correlations were found with age, time since diagnosis, cramps, or other mobility parameters.

**Conclusions** Fatigue is a common symptom among individuals with ALS and may be present in all stages of the disease. This symptom was correlated with worse functioning, poorer QoL, greater pain intensity, disease severity, muscle weakness, and the female sex in individuals with ALS.

## Resumo

**Antecedentes** Esclerose lateral amiotrófica (ELA) é uma doença neurodegenerativa fatal que leva à fraqueza muscular e paralisia. Fadiga é um sintoma incapacitante, frequentemente relatado na ELA, mas permanece pouco investigado nessa população. Assim, uma investigação acurada deste sintoma e dos possíveis fatores associados é necessária para auxiliar em uma abordagem terapêutica adequada.

**Objetivo** Investigar a presença de fadiga em indivíduos com ELA e possíveis fatores correlacionados a este sintoma.

**Métodos** Participaram 65 indivíduos com ELA esporádica. Aspectos demográficos, clínicos e funcionais foram investigados. Foram utilizadas: Escala de Severidade de Fadiga (FSS, na sigla em inglês), Escala Funcional de ELA (ALSRFS-R, na sigla em inglês) e questionário de Qualidade de Vida (QV) (ALSAQ-40, na sigla em inglês). Análises descritivas e de correlação foram realizadas no programa SPSS para Windows versão 19.0 (IBM Corp., Armonk, NY, USA).

**Resultados** Dos 65 indivíduos avaliados, 44,6% ( *n* = 29) apresentaram fadiga pela FSS. A intensidade da fadiga foi 5,4 ± 1,2 e apenas 10,4% usavam medicamento específico para fadiga. Diferenças entre os grupos com e sem fadiga foram encontradas em relação ao sexo ( *p* = 0,049), intensidade da dor ( *p* = 0,026), funcionalidade ( *p* = 0,004), gravidade da doença ( *p* = 0,029) e QV ( *p* = 0,000). A fadiga foi correlacionada com a intensidade da dor (r = 0,425; *p* = 0,001), força muscular (r = - 0,356; *p* = 0,004), funcionalidade (r = - 0,363; *p* = 0,003) e QV ( *r* = 0,481; *p* = 0,000). Não foram encontradas correlações com idade, tempo de diagnóstico, cãibras ou outros parâmetros de mobilidade.

**Conclusões** A fadiga é um sintoma comum entre os indivíduos com ELA e pode estar presente em todos os estágios da doença. Este sintoma estava correlacionado à pior funcionalidade, pior QV, gravidade, intensidade da dor, fraqueza muscular e sexo feminino em indivíduos com ELA.

## INTRODUCTION

Amyotrophic lateral sclerosis (ALS) is a degenerative motor neuron disease characterized by the progressive impairment of upper and lower motor neurons. 1 2 Epidemiological studies in the literature on ALS report an incidence of 2 to 3 cases in every 100,000 individuals. 2 Most affected individuals (90%) have sporadic ALS, which is of an unknown origin, and only 10% of cases are of a familial origin. 2 The prognosis is poor, as most patients die within an average of 3 to 5 years after the onset of symptoms, 3 with respiratory failure the most common cause of death. 1 3

As the disease progresses, generalized muscle weakness and fatigue occurs, which leads to the loss of movements and a diminished capacity to perform activities of daily living. Besides motor impairment, extramotor manifestations can also occur, such as cognitive and behavioral disorders. 1 2 Fatigue is a disabling symptom often reported in ALS. 4 5 However, few studies have investigated fatigue in this population.

The etiology of fatigue in ALS is not fully understood but appears to be multifactorial. The proposed mechanisms include muscle activation disorder, muscle changes resulting from disuse, cardiorespiratory deconditioning, and psychological factors. 6 7 8 Fatigue is generally perceived in two ways: general fatigue (sensation of weariness in the entire body) and physical fatigue, which is related to muscle use (reversible motor weakness). 6 9

One characteristic of fatigue in ALS is that the manifestation of weariness or exhaustion is only partially relieved by rest and tends to worsen throughout the day, 6 8 9 affecting physical performance and disabling the individual. 6 10 Therefore, fatigue has been associated with functional limitations and restrictions with regards to family/social participation, which can lead to depression and exert a negative impact on the perception of quality of life of the individual (QoL). 4 9 10 11

Despite the impact of fatigue on the lives of individuals with ALS, little is known regarding the possible clinical and functional factors related to its presence, 6 8 12 which hampers the implementation of prevention and treatment measures. Therefore, the aim of the present study was to investigate the presence of fatigue in individuals and possible clinical/functional factors correlated with its occurrence.

## METHODS

### Study design

An exploratory cross-sectional study was conducted.

### Sample

The present study involved a convenience sample of 65 individuals with a diagnosis of sporadic ALS according to the Awaji criteria 13 in care at the neuromuscular disease outpatient clinic of the university hospital affiliated with the Universidade Federal de Minas Gerais (UFMG, in the Portuguese acronym) in 2019. Patients who had diagnosis of familial ALS, flail arm and flail leg variants, other motor neuron disease (progressive muscular atrophy, primary lateral sclerosis), signs and symptoms of dementia screened by the medical team or medical history of other neurological disease were excluded to ensure a more homogenous group. The present study received approval from the UFMG institutional review board (number: 08661019.9.0000.5149).

#### *Sociodemographic and clinical characteristics*

A questionnaire was used to collect data on sociodemographic and clinical characteristics: age, sex, educational status, comorbidities, medication use, fatigue medication, nonpharmacological treatment for fatigue, hospitalization, ventilation, gastrostomy, pain in the previous week, mobility (go from sitting to standing, change positions in bed, maintain standing position and walking capacity), use of mobility aids, multidisciplinary approach, time in years since ALS diagnosis, site of onset of symptoms, and use of riluzole.

#### *Fatigue assessment*

Fatigue was assessed using the Fatigue Severity Scale (FSS), which is a nine-item self-report questionnaire used to investigate the severity of fatigue in the daily life of the respondent. Each statement on the scale is scored from 1 (strongly disagree) to 7 (strongly agree) points. The total is calculated by the average of all items and a score ≥ 4 indicates the presence of fatigue. 14

#### *Functional assessment - ALS specific questionnaire*

The Amyotrophic Lateral Sclerosis Functional Rating Scale-Revised (ALSFRS-R) was used for the functional assessment. The ALSFRS-R questionnaire uses an ordinal scale to assess capacity and independence on 12 functional activities (speech, salivation, swallowing, handwriting, handling utensils and cutting food, dressing and personal hygiene, turning in bed and adjusting bed clothes, walking, climbing stairs, dyspnea, orthopnea, and breathing insufficiency). Each function is scored from 0 to 4 points and the total ranges from zero (severe dysfunction) to 48 (normal). 15 The ALSFRS-R score was categorized in three stages of severity: mild (37 to 48 points), moderate (25 to 36 points) and severe (0 to 24 points). 16 17

#### *Muscle strength assessment*

Muscle strength was measured bilaterally and scored on a scale from 0 to 5 points using the Medical Research Council (MRC) scale. Four upper limb muscles (wrist extension, wrist flexion, elbow flexion and shoulder abduction) and four lower limb muscles (ankle dorsiflexion, plantar flexion, hip extension and flexion) were considered. The score of the 16 muscle groups produced an overall strength score ranging from 0 to 80 points, 18 19 20 with a higher overall score denoting greater muscle strength.

#### *Quality of life assessment*

Quality of life was assessed using the ALS Assessment Questionnaire-40 (ALSAQ-40), which is specifically designed to evaluate the QoL of patients with ALS. The AALSAQ-40 addresses five domains that are normally compromised in motor neuron diseases: food, communication, activities of daily living and independence, mobility, and emotional aspects. The score for each domain ranges from 0 to 100. The total score is determined from the sum of the domains and ranges from 0 to 500, with higher scores denoting worse QoL. 21

#### *Statistics*

Descriptive analysis was performed with the calculation of frequency or measures of central tendency and dispersion, depending on the characteristics of each variable. The Shapiro-Wilk test was used to determine the normality of the data. For the comparison of subgroups according to the presence of fatigue (group with and without fatigue), the multivariate analysis of variance (MANOVA), the Mann-Whitney test, the chi-squared test, or the Fisher exact test was used depending on the variable analyzed. In order to reduce the occurrence of type 1 error, comparisons of quantitative dependent variables were performed using MANOVA. Considering the study design had only two factors, the post hoc analyzes were automatically presented by the software via an independent T-Student test. Either the Pearson or the Spearman correlation test was used to investigate correlations between fatigue and clinical/functional variables, depending on the normality of the data. For the correlation analysis, the variables (age, time since diagnosis, pain intensity, overall muscle strength, functional capacity, and QoL) were selected when presenting significant differences between the groups with and without fatigue or due to their clinical relevance. All analyses were performed with the aid of IBM SPSS Statistics for Windows version 19.0 (IBM Corp., Armonk, NY, USA). The significance level was set at 5% ( *p* < 0.05).

## RESULTS

Among the total of 65 individuals evaluated, 44.6% ( *n* = 29) had fatigue based on the screening test (FSS). The mean reported fatigue intensity was 5.4 (SD = 1.2) and only 10.4% ( *n* = 3) of the individuals with fatigue took specific medications for controlling the condition (bupropion and amantadine). No participants reported undergoing any specific nonpharmacological treatment for controlling fatigue.

The group that reported fatigue was predominantly composed of women (51.7%) and limb onset of ALS (72.4%), with a median of 2 years since the diagnosis (range: 0.7 to 20 years). The clinical and demographic characteristics of the groups with and without fatigue are displayed in Table 1 . Significant differences between groups were found regarding sex ( *p* = 0.049), pain intensity ( *p* = 0.026), total ALSFRS-R functional scale score ( *p* = 0.004), level of severity of ALS ( *p* = 0.029), and QoL measured using the ALSAQ-40 ( *p* = 0.000). No significant differences were found for the other variables ( Table 1 ).

**Table 1:** Demographic and clinical characteristics of ALS participants

Characteristics *n* (%) or *mean (SD)* or *median (min-max)*			Fatigue *n* = 29 (44.6%)	Without fatigue *n* = 36 (55.4%)	*p-value*
**Age** (years old)			57.8 (9.9)	55.6 (11.9)	0.427
**Male**			14 (48.3%)	26 (72.2%)	0.049*
**Educational level** (years)			3.9 (1.1)	3.9 (1.2)	0.873
**Time since diagnosis** (years)			2 (0.7–20)	3 (0.6–19)	0.290
**Site of onset**	Upper limb		08 (27.6)	11 (30.6)	0.310
	Lower limb		13 (44.8)	23 (63.9)	
	Bulbar		08 (25.4)	02 (5.6)	
**Fatigue intensity** *(FSS score)*			5.4 (1.2)	16 (0.8)	0.000*
**Pain**			19 (65.5%)	18 (50%)	0.209
**Pain intensity**			4 (0 - 10)	0 (0 - 8)	0.026*
**Comorbidity**			19 (65.5%)	17 (47.2%)	0.140
**Medication**			27 (93.1%)	31 (86.1%)	0.366
**Riluzole use**			21 (72.4%)	25 (69.4%)	0.794
**Fatigue medication**			3 (10.4%)	0	–
**Nonpharmacological treatment for fatigue**			0	0	–
**Noninvasive ventilation**			6 (20.6%)	6 (16.7%)	0.982
**Tracheostomy/mechanical ventilation**			0	0	0
**Gastrostomy**			5 (17.2%)	2 (5.6%)	0.131
**Hospitalization in last year**			10 (34.5%)	10 (27.8%)	0.560
**Mobility**	Sit to stand	*Able without assistance*	6 (20.7%)	11(30,6%)	0.722
		*Able with assistance*	15 (51.7%)	18 (50.0%)	
		*Unable to sit to stand*	8 (27.6%)	7(19.4%)	
	Ambulatory capability	*Able without assistance*	9 (31.0%)	15 (41.7%)	0.703
		*Able with assistance*	10 (34.5%)	13 (36.1%)	
		*Unable to walk*	10 (34.5%)	8 (22.2%)	
**Mobility aids**			19 (65.5%)	22 (61.1%)	0.714
**ALSFRS-R total**			25.8 (11.1)	33.4 (9.7)	0.004*
**Stages/levels of severity**	Mild		6 (20.7%)	19 (52.8%)	0.029*
	Moderate		11 (37.9%)	9 (25.2%)	
	Severe		12 (41.4%)	8 (22.2%)	
**Muscle strength**			38.6 (14.9)	42.5 (17.1)	0.332
**QoL (ALSAQ-40)**			319.8 (103.03)	219.1 (112.9)	0.000*

Abbreviations: ALSAQ-40, Amyotrophic Lateral Sclerosis Assessment Questionnaire-40; ALSFRS-R, Amyotrophic Lateral Sclerosis Functional Rating Scale-Revised; FSS, Fatigue Severity Scale; min-max, minimum-maximum; n, number; SD, standard deviation.

Note: ^*^ statistically significant difference.

Significant correlations, but fair according to Portney et al., 22 were found between fatigue intensity and functioning measured using the ALSFRS-R (r = - 0.363; *p* = 0.003), QoL (r = 0.481; *p* = 0.000), pain (r = 0.425; *p* = 0.001) and muscle strength (r = -0.356; *p* = 0.004). No significant correlations were found for the other variables (age and time since diagnosis). Correlations between fatigue intensity and clinical/functional factors are displayed in Table 2 .

**Table 2:** Correlation between fatigue and clinical/functional variables

Variables	Fatigue	
	r	*p-value*
**Age**	0.1	0.427
**Time since diagnosis (years)**	- 0.040	0.753
**Functioning (ALSFRS-R)**	- 0.363	0.003*
**QoL (ALSAQ-40)**	0.481	0.000*
**Pain intensity**	0.425	0.001*
**Muscle strength**	- 0.356	0.004*

Abbreviations: ALSAQ-40, Amyotrophic Lateral Sclerosis Assessment Questionnaire-40; ALSFRS-R, Amyotrophic Lateral Sclerosis Functional Rating Scale-Revised; QoL, quality of life.

## DISCUSSION

Fatigue is a prevalent symptom among individuals with ALS. Clinically, fatigue is correlated with worse functional capacity, muscle weakness, and poorer QoL, but remains an underexplored symptom in studies and clinical practice.

In the present study, 44.6% of the individuals presented fatigue. The frequency of fatigue in individuals with ALS reported in the literature varies greatly. Some studies report similar frequencies to that found in the present investigation, such as 44% 11 and 52.7%, 12 whereas other report higher frequencies, such as 83% 8 and 90%. 23 McElhiney et al. 11 and Lo Coco et al., 12 who found similar frequencies, also used the FSS to screen for fatigue and the participants had similar clinical characteristics in terms of the time since diagnosis and ALSFRS-R score. Ramirez et al. also used the FSS but found a much higher frequency of fatigue (83%). One explanation for this divergence may be the large difference in the time since diagnosis, the median of which was 42 months in the study by Ramirez et al. 8 compared with 24 months in the present investigation. In the study by Nicholson et al., 23 who found that 90% of the participants had fatigue, time since diagnosis was longer (mean: 59.9 ± 55 months) and the authors did not use a specific fatigue assessment tool, merely asking the participants whether they had the symptom. Therefore, the difference in the frequency of fatigue among studies may be related to the type of instrument used to screen for the symptom and/or the characteristics of the sample. Future studies should investigate changes in the occurrence of fatigue throughout the course of the disease, as time since diagnosis seems to exert an influence on the perception of the symptom.

In the present study, a significant difference was found between the sexes, with fatigue occurring less in men than women. This difference in prevalence between the sexes has also been found in the general population without a diagnosis of ALS. 24 According to the literature, the possible explanations for this difference in the perception of fatigue may be the central component of the symptom, as women are more likely to present neuropsychological alterations, and issues related to hormonal variations. 24 However, there is no consensus on the mechanism of fatigue and differences in the perception of the symptoms between men and women. Further studies are needed to investigate this issue better.

A fair relationship (r = 0.425) was found between fatigue intensity and pain intensity in the present study. Despite being frequent symptoms and often overlooked in clinical practice, little investigation has been made regarding the relationship between these symptoms. 17 25 Both pain and fatigue occur during the course of the disease and are associated with a worse QoL as well as with the occurrence of depression. 12 17 25 Fatigue and pain in ALS seem to follow a similar pathophysiological pathway, as both are involved in a modulation of the central component and have manifestations that involve the overlapping of emotional, cognitive, and physical symptoms. 6 7 8 9 Hence, future studies should investigate the relation between pain and fatigue better to identify whether these symptoms influence each other directly or indirectly or share the same pathophysiological pathway.

Muscle weakness was correlated with fatigue in the present sample. We found an inverse relationship between fatigue intensity and muscle strength measured by the MRC scale, and a correlation coefficient of - 0.356. This finding may be related to peripheral mechanisms (associated with muscle strength dysfunction) that contribute to fatigue in ALS. 12 Affected individuals normally make efforts to maintain capacity and functional performance, as the death of motor neurons leads to weakness and progressive muscle atrophy. In contrast, Ramirez et al. 8 found no correlation with fatigue, despite finding muscle weakness in the majority of individuals in the sample. The authors justified this lack of correlation by the fact that the sample was composed of individuals with a good functional level, 8 which differs from the sample in the present study.

Fatigue was inversely correlated with functioning measured using the ALSFRS-R functional scale (worse functioning was found among individuals with a higher fatigue score). Previous studies have also reported that more advanced stages of the disease (worse scores on the functional scale) are associated with higher rates of fatigue. 11 12 Lo Coco et al. 12 also found that the severity of ALS is the main predictor of fatigue in this population. Generalized muscle weakness, disuse, and physical deconditioning, which worsen as the disease progresses, may contribute to this finding. In contrast, Gibbons et al. 26 and Ramirez et al. 8 found no correlation between the ALSFRS-R score and fatigue. It is likely that the characteristics of the sample in these studies, such as time since diagnosis and the heterogeneity of the sample, may have influenced the results. As divergences are found in the literature regarding the relation between functional capacity and fatigue, future studies should investigate possible factors that influence this relation.

Fatigue was related to poorer QoL in the present sample (r = 0.481). This finding was expected and has been widely described in previous studies. 4 9 10 Fatigue is a limiting symptom that restricts social participation and exerts a negative impact on family life, which can impact one's perception of QoL. 6 Gibbons et al. 9 highlight the multifactorial nature of fatigue, which is expressed not only in muscle weakness but also in central factors, such as the perception of a lack of energy, exerting a negative impact on aspects related to QoL. As a disease with no cure and few effective therapeutic possibilities, improving the QoL of individuals with ALS becomes the central care goal. It is therefore important to investigate the presence of fatigue in this population and implement intervention strategies in clinical practice with the aim of improving the perception of QoL. 9

Despite the negative impact of fatigue, especially in individuals with ALS, the clinical management of this symptom is scarce. 6 In the present study, only 10.4% of the individuals took medication for the treatment of fatigue and none reported undergoing nonpharmacological therapy for the treatment of this symptom. For interventions to be effective, a multimodal approach to fatigue is needed and this symptom should always be investigated during clinical follow-up. 6 12

The present study has limitations that should be considered, such as the cross-sectional design, the sample composed of patients from a specialized center and the use of a unidimensional instrument for the assessment of fatigue. Longitudinal studies are needed to gain a better understanding of factors associated with fatigue and enable the determination of causality between variables. Moreover, future studies should use assessment tools to investigate the physical and mental components of fatigue independently and thus identify factors associated with central and peripheral fatigue.

In conclusion, the present study verified that fatigue was correlated with worse functioning, poorer QoL, greater pain intensity, disease severity, muscle weakness and the female sex in individuals with ALS. Despite being frequent, fatigue is underinvestigated in clinical practice and scientific research involving this population. Considering the prevalence, complexity and consequences of fatigue, further studies are needed to investigate this symptom better and enable more adequate treatment and therapeutic options.
